# Fast design of arbitrary length loops in proteins using InteractiveRosetta

**DOI:** 10.1186/s12859-018-2345-5

**Published:** 2018-09-24

**Authors:** William F. Hooper, Benjamin D. Walcott, Xing Wang, Christopher Bystroff

**Affiliations:** 1 Emmes Corporation, Rockville, Washington, MD USA; 2 Department of Biology, Rensselaer Polytechnic Institute, Troy, NY USA; 3 Department of Chemistry and Chemical Biology, Rensselaer Polytechnic Institute, Troy, NY USA; 4 Department of Computer Science, Rensselaer Polytechnic Institute, Troy, NY USA

**Keywords:** Indel, Bystroff, InteractiveRosetta, Rosetta, PyRosetta, T7 endonuclease I, Protein design, Simulation, Loop modeling

## Abstract

**Background:**

With increasing interest in *ab initio* protein design, there is a desire to be able to fully explore the design space of insertions and deletions. Nature inserts and deletes residues to optimize energy and function, but allowing variable length indels in the context of an interactive protein design session presents challenges with regard to speed and accuracy.

**Results:**

Here we present a new module (INDEL) for InteractiveRosetta which allows the user to specify a range of lengths for a desired indel, and which returns a set of low energy backbones in a matter of seconds. To make the loop search fast, loop anchor points are geometrically hashed using C *α*-C *α* and C *β*-C *β* distances, and the hash is mapped to start and end points in a pre-compiled random access file of non-redundant, protein backbone coordinates. Loops with superposable anchors are filtered for collisions and returned to InteractiveRosetta as poly-alanine for display and selective incorporation into the design template. Sidechains can then be added using RosettaDesign tools.

**Conclusions:**

INDEL was able to find viable loops in 100% of 500 attempts for all lengths from 3 to 20 residues. INDEL has been applied to the task of designing a domain-swapping loop for T7-endonuclease I, changing its specificity from Holliday junctions to paranemic crossover (PX) DNA.

**Electronic supplementary material:**

The online version of this article (10.1186/s12859-018-2345-5) contains supplementary material, which is available to authorized users.

## Background

Computational protein design is the task of finding an energy-optimal amino acid sequence for a backbone structure. Simplifying assumptions, such as fixed backbone atoms and discrete side chain conformations [1, 2], have been necessary because of the prohibitive size of the computational sequence search space. But, as computational resources improve, simplifying assumptions are falling away in favor of increased accuracy [3]. No longer is the backbone assumed to be fixed [4], and side chain conformations are no longer assumed to fall into discrete distributions [5]. The design process is increasingly looking like the natural process of random mutation and energetic selection. But we still assume that the template does not undergo deletions or insertions. To make protein design even more like molecular evolution, we should allow the algorithm to explore the space of insertions and deletions (indels).

Searching the space of indels presents a host of computational problems. The expanded search space now includes the locations of the ‘anchor’ residues, defined as the last residue before and the first residue after the indel. Additionally, the length is variable, as is the sequence. Out of the necessity for computationally efficiency, we propose a hierarchy of searches. When indels occur naturally, they create a mutational “hotspot” around the gap position. This results in a viable but energetically suboptimal species immediately after indel introduction, increasing the probability of energetically advantageous mutations. If we want our algorithm to follow this natural process, our first step should be to explore the space of loop lengths without considering the side chains. This is the problem we address in this paper. The related problems of searching backbone flexibility and side chain mutation space are already solved by existing algorithms for energy minimization and protein design [6, 7], respectively. If we are justified in separating the indel search from the sequence design, then we may be able to open up a new world of protein design in which the chain length is now a variable.

Current approaches to loop modeling are either physics-based or template based. The physics-based algorithms include kinematic closure (KIC), fragment assembly and analytic loop closure (FALC), molecular dynamics (MODELLER), and many more [8–11]. KIC was inspired by a technique in robotics for positioning joints with constraints. Random loop subfragments are selected to define 6 pivot points, then values for the 6 pivots are solved such that the loop is closed. KIC is usually used in the context of a Monte Carlo algorithm with simulated annealing [8, 9]. FALC is a hierarchical approach that employs KIC. Database fragments are found for 5 and 7 amino acid residue segments. These are inserted using KIC, then scored and ranked using a force field. Rotamers are added and the fragments are again scored and ranked [10]. In contrast, MODELLER [11] randomizes the loop atomic positions, then uses all-atom energy minimization and molecular dynamics to predict the conformation, but this method is CPU intensive. Other notable methods include GalaxyLoop [12, 13], RAPPER [14, 15], and PLOP/HLP [16–18].

INDEL is a template-based loop design algorithm that draws loops from a list of high-resolution crystal structures precompiled into a random-access database. Loops are indexed by anchors using C *α*-C *α* and C *β*-C *β* distances, and the two-dimensional distance bins are sorted and mapped to a second-level index which can be calculated directly from the anchor point C *α*-C *α* and C *β*-C *β* distances. This two-level look-up approach allows for fast retrieval without distance calculations and without searching the database. Candidate loops are pruned in a second pass if backbone collisions are found, and in a third pass the remaining candidate loops are energy minimized and scored using Rosetta. The final candidates may be used as templates for design using fixbb or other Rosetta-Design protocols.

As proof of concept, we have applied INDEL to a comparative modeling case in which a two-residue insertion was made in the core of green fluorescent protein, and the structure was subsequently solved by X-ray crystallography (AT-GFP, PDB 4LW5) [19]. The algorithm quickly identified a database loop that closely matched the experimentally determined one. We also show that INDEL can be applied to a system that contains multiple chains, protein and DNA together, and a system which contains homo-dimeric symmetry, where two copies of the loop are designed simultaneously.

## Methods

### Database structure

The loop database structure is inspired by the constant-time speed and key-value access of a hash table. Here, three keys are used to access loops: the distance between loop anchor C *β*’s (Å), anchor C *α*’s (Å), and loop length (residues). Matching each of these two distances assures that the anchor residues of a loop are both the right distance apart and are in the right relative orientation.

A goal of many hash table implementations is to avoid “collisions”, where multiple keys map to the same location in the table. However, in this case, collisions are simply many database loops that map to the same anchor positions; here we want to retrieve them all. Allowable distances range from 0 to 50 Å, with a resolution of 0.1 Å. The fine-grained binning of loops allows the program to dynamically control the number of loops returned.

The first step in constructing the loop database was to build a repository of protein structures. Coordinates were drawn from the Top8000 dataset, a curated set of 8000 high-quality crystal structures whose purpose was to update the MolProbity software [20].

Each residue was reduced to a 70-byte binary record containing PDB ID, chain, residue type, residue number, and coordinates for the atoms N, C *α*, C, O, and C *β*. Residues were renumbered sequentially to avoid complications due to insertion numbering. When a glycine was encountered, a C *β* position was calculated using Kabsch’s algorithm [21]. All residues from all proteins were concatenated into a single, random access file (file “C”, pdblist.dat,128.5 MB).

An additional two random access files were constructed to perform the look-up. The first (file “A”, grid.dat, 40 MB) is a three dimensional array, 500x500x20 in size, where the axes correspond to C *αCα* distance, C *βCβ* distance, and the anchor separation distance. Each entry in the array is a tuple: a pointer to a record in file B, and the total number of contiguous records starting from that one. The second database file (file “B”, looplist.dat, 271.4 MB) consists of tuples: a pointer to the beginning of a loop in file C, and the loop’s length in residues. These files are akin to a library’s card catalogue where each drawer of the catalogue represents a pair of C *αCα* and C *βCβ* distances. Inside each drawer of this catalogue are twenty cards indicating where loops of a desired length can be found for those distances. A similar hashing scheme exists in Rosetta’s LoopHash protocol, where the PDB was broken into fragments and hashed according to a 6-dimensional rigid body transform required to superimpose one anchor residue on the other [22].

File B was created from file C by iterating over record numbers for all intra-chain anchor pairs with separation distances from 3 to 19, and sorting them by C *αCα* distance, C *βCβ* distance, and separation. File A was created from file B by reading and counting the number of records in bins of width 0.1Å in C *αCα* distance and C *βCβ* distance and bins of width 1 in sequence separation. Finally, file A was populated at each grid point with the file B record number for the start of a list of contiguous file C records, along with the length of that list.

### Database lookups and loop insertion

A full walk-through of the INDEL loop design process is provided in Supplementary Data. To begin an INDEL database lookup from within InteractiveRosetta [23], the user first sets constraints for the search. Specifically, anchor residues are chosen, a range of allowable loop lengths, the minimum and maximum number of results to return. INDEL pull loops from the database and superposes the anchor coordinates. Loops are immediately rejected if they do not superimpose better than an RMSD cutoff, if they collide with the target structure backbone atoms, or if they are structurally redundant with respect to earlier results in the search. INDEL writes out the search results, which are subsequently inserted via PyRosetta’s AnchoredGraftMover module. Each completed model is ranked by the Rosetta scoring function, and the top candidates are returned to the user for viewing. Upon selection of a loop, the side chains may be designed using the Protein Design (Rosetta’s Fixbb) protocol, and the energy may be minimized using the Energy Minimization protocol.

## Results

### Timing

**Fig. 1:**
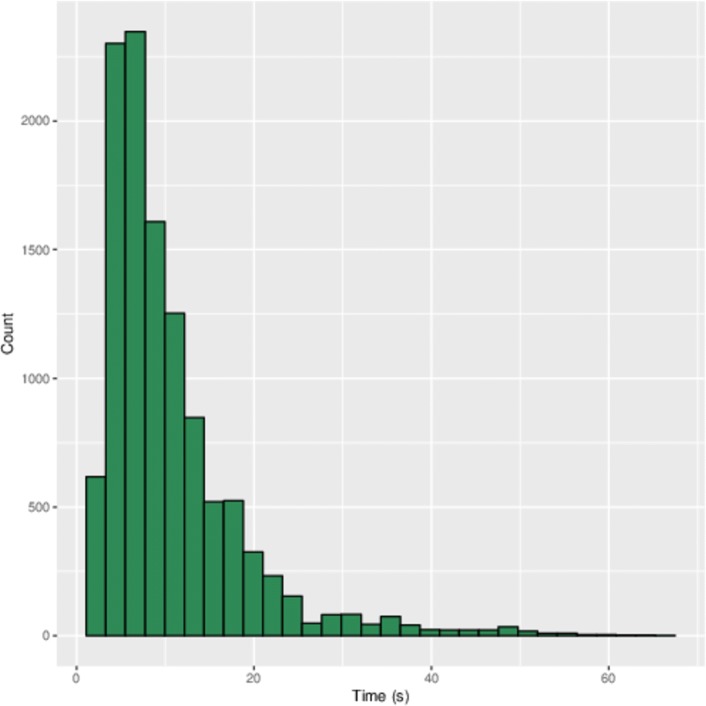
INDEL insertion times. This histogram depicts the amount of time spent on each loop insertion for scaffolds of varying size via INDEL. The vast majority (75th percentile) of loop insertions occur within 13 s

Fast retrieval of viable loop coordinates is essential for an interactive modeling program, and the program must run reasonably fast on a standard laptop with as few as one CPU. Hashed retrieval of loops is a fast, constant-time lookup, since no search is taking place. Most of the delay comes from the need to calculate distances between loop and target atoms, which follows a low-order polynomial (*O*(*n*^2^)). Further delay may depend on the location of the loop, since a more crowded environment would entail testing more loops to find one with no collisions. But in benchmarking the code using a variety of loop length, we found the loops were returned in under 13 s in the vast majority of cases, and never did it take over 70 s to return an answer, regardless of length or location (Fig. 1).

### Native length loop reconstruction

**Fig. 2:**
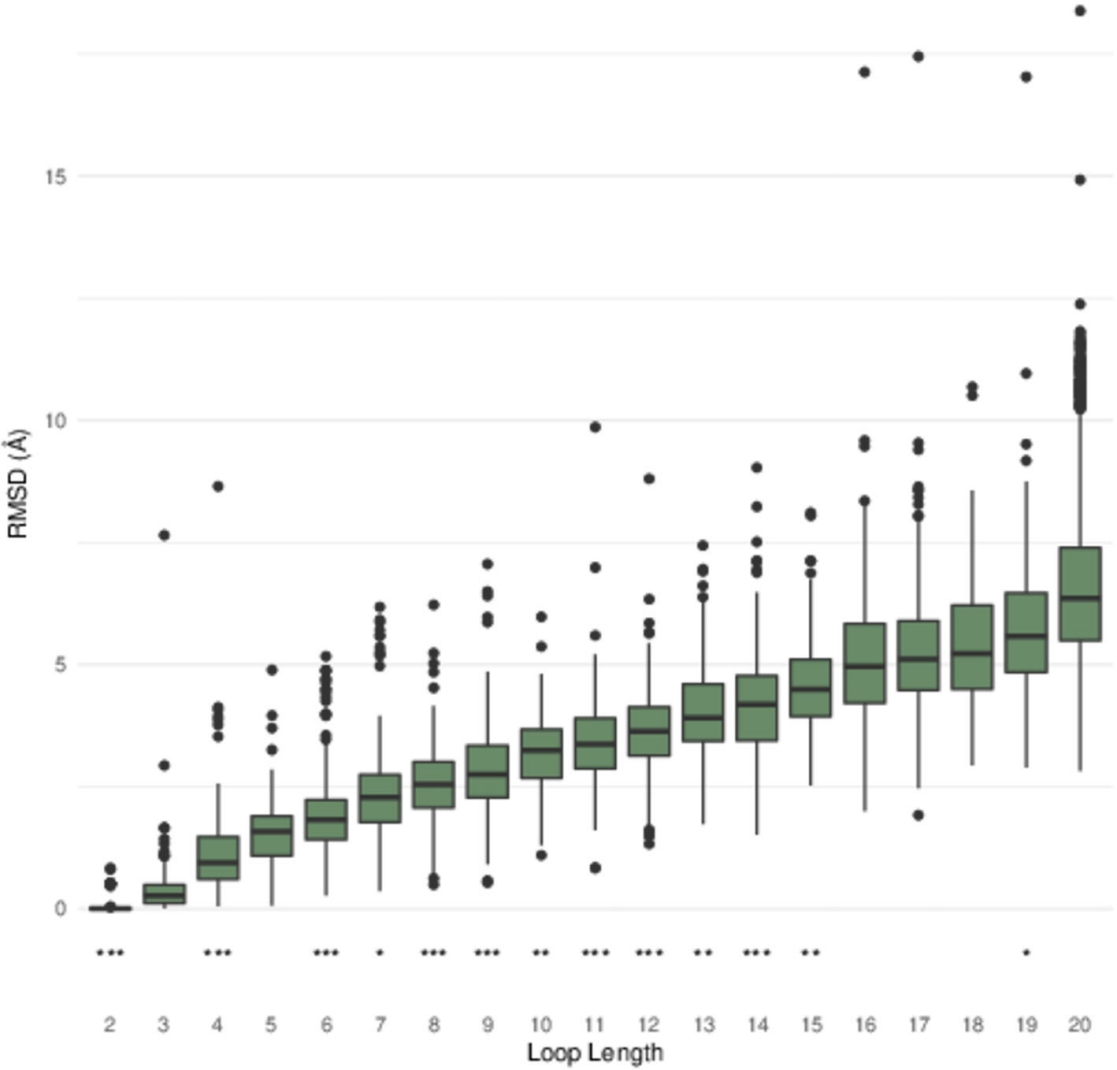
Loop reconstruction performance. For loops of length 2–20 residues, 500 runs of INDEL were performed on randomly selected positions of database proteins that were all random coil (i.e. not helix or strand) positions. The distribution of the 500 RMSDs is expressed as a box plot, with outliers plotted as dots. Below each box plot is the significance of the collision check as a predictor of low RMSD, as measured using ROC ∗∗∗=*p*<0.001,∗∗=*p*<0.01,∗=*p*<0.05

**Fig. 3:**
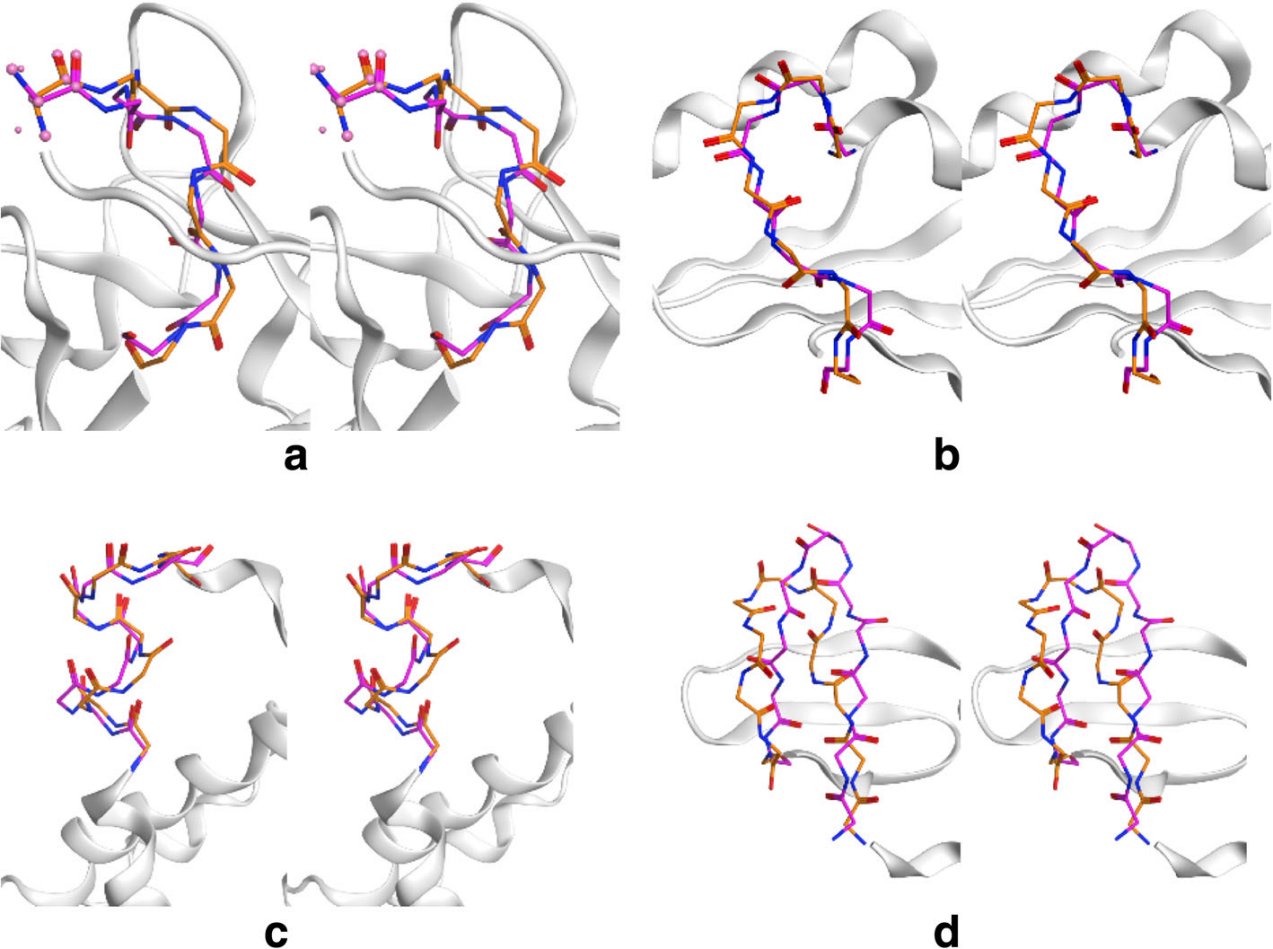
Example loop reconstructions. Stereo images show loops of native length compared to the native loop for lengths **a** 5 (1jlx 91–95), **b** 7 (1eay 214–220), **c** 9 (3gqb 465–473), and **d** 11 (1s72 37–47). Native loops are in orange. Designed loops in purple

INDEL is capable of inserting loops of lengths between 2 and 20 residues long. For each of these 19 lengths, a random loop region of the same length was selected for INDEL design from a random protein within the VAST nr-PDB database [24, 25]. The RMSD of the inserted loop to the original loop was then assessed. The loop was rejected if the shortest distance between a backbone atom of the inserted loop and a backbone atom of the target protein was below INDEL’s collision cutoff (4.0 Å by default). All loops, whether accepted or rejected, were sorted by the backbone atom RMSD to the native loop and the ROC curve was calculated [26, 27] to assess the ability of the algorithm to preferentially keep low-RMSD loops. The *p*-value is the probability of getting the ROC value or better after scrambling the data. Accepted loops were sorted by RMSD and the distributions are summarized in Fig. 2. Lowest-RMSD examples are often within 1Å RMSD (Fig. 3).

### Modeling an engineered insertion.

**Fig. 4:**
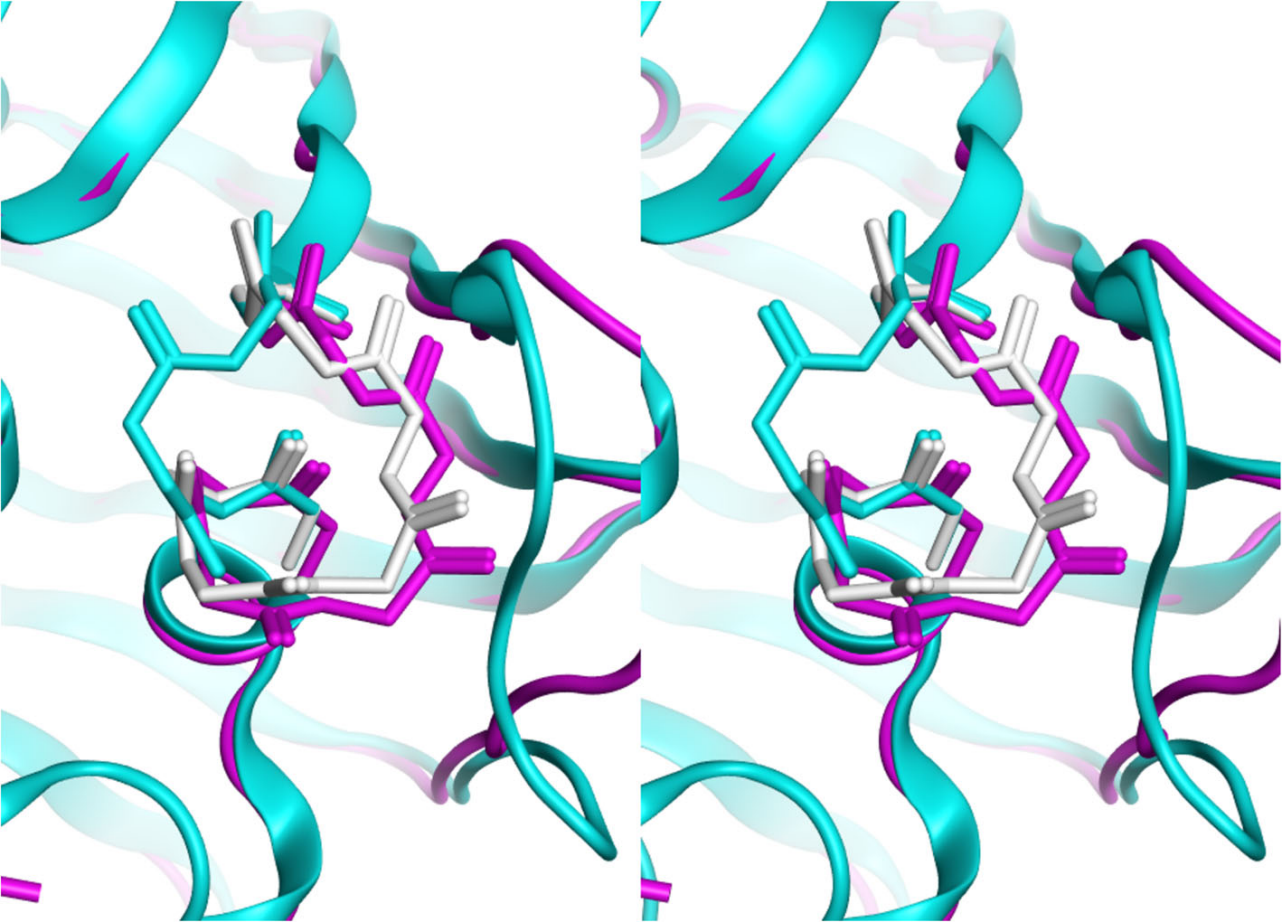
AT loop reconstruction. Stereo image showing superfolder GFP near the 88-MP-89 cis-peptide bond (cyan ribbon, bonds). Into the wild-type template a 6-residue loop was inserted using INDEL. The lowest RMSD resulting loop (white) closely matches the experimentally determined structure of “All-trans” GFP (magenta)

INDEL was used to model a loop that was engineered into GFP, converting a loop containing a cis peptide bond to a 2-residue longer loop that has all trans peptide bonds. The variant, called All-trans-GFP or AT-GFP, was subsequently solved by X-ray crystallography (PDBid 4LW5) [19]. The algorithm quickly identified a database loop that closely matched the experimentally determined one. Figure 4 shows the original structure, the X-ray structure of the variant, and the loop predicted by INDEL.

### Designing a linker for a domain-swapped dimer.

**Fig. 5:**
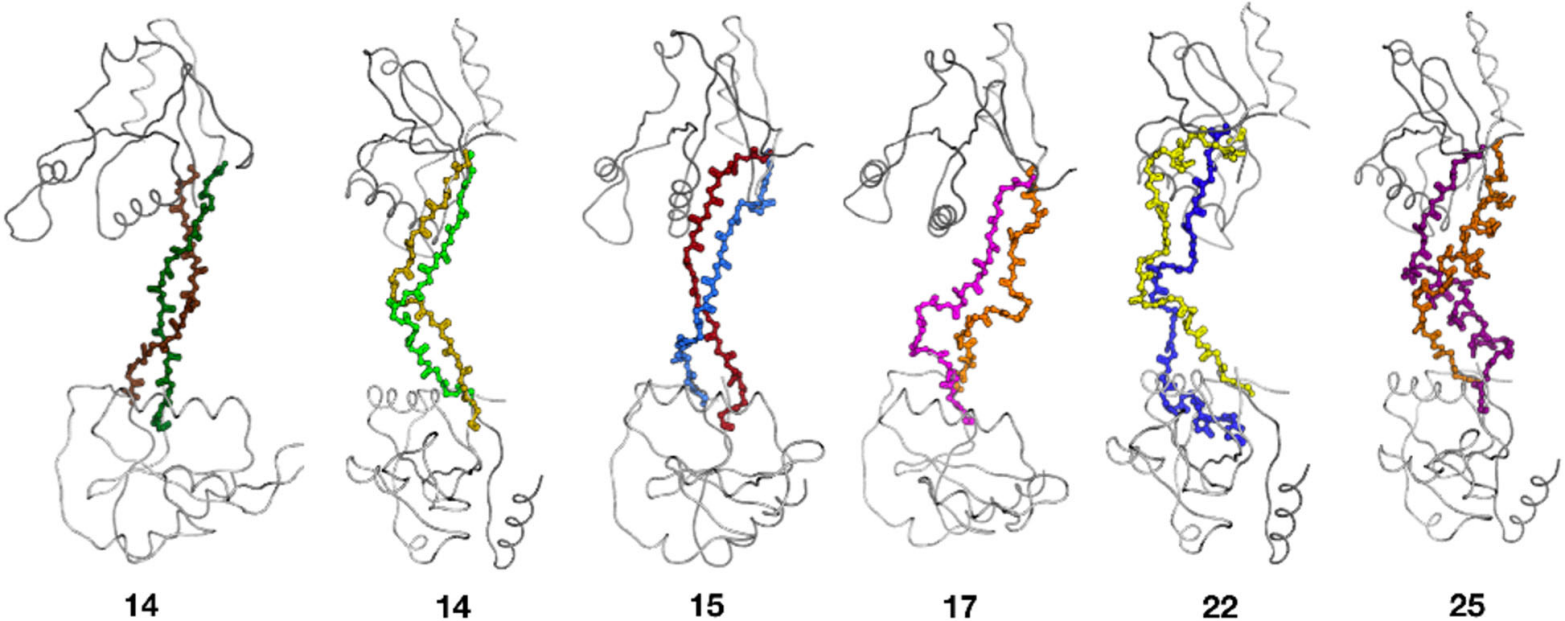
T7 endoI models. T7 endonuclease I is a dumbbell-shaped, domain-swapped dimer. INDEL was used in symmetrical dimer mode to find loops of various length that would connect the two globular domains. Each was energy minimized before making this figure

INDEL has been used in this lab to design linkers between globular domains. The enzyme T7 endonuclease I (T7 endoI) cuts DNA at Holliday junctions (HJ) [28, 29], but our desire is to design a version of the enzyme that cuts paranemic crossover (PX) DNA [30]. The latter is a DNA tetraplex that has unique distances and orientations between fissile phosphate backbone positions. If T7 endoI could be engineered to have the correct spacing and orientation between its two binding sites, then the enzyme specificity could be optimized to recognize PX instead of HJ. Figure 5 shows the results of loop design. In this case, two-fold symmetry was generated for each result of the loop search tom complete domain-swapped homo-dimer structure. Two-fold symmetry was enforced during the subsequent collision checking but not during energy minimization (energy minimization is not part of the INDEL protocol).

## Discussion

**Fig. 6:**
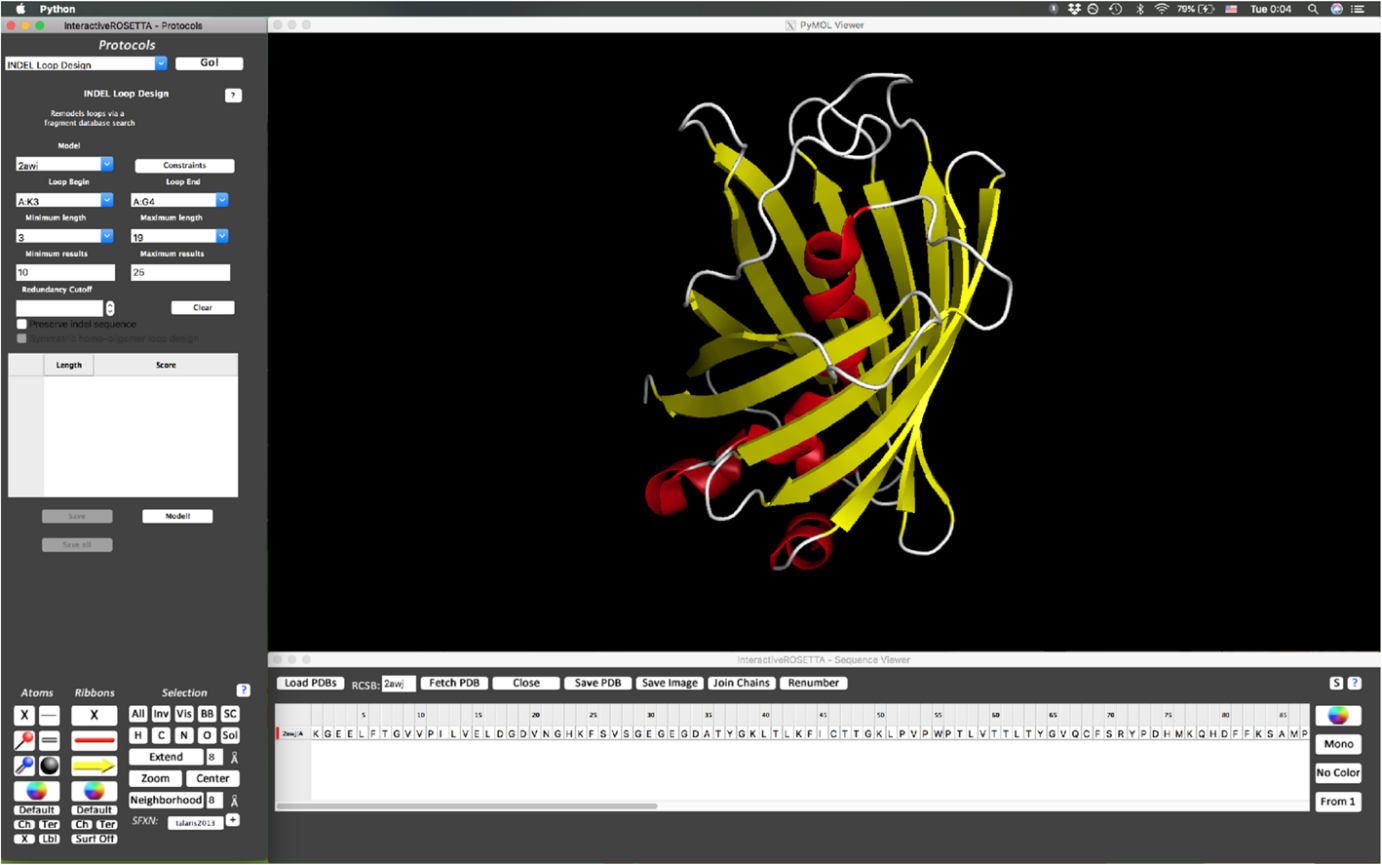
INDEL design window. An example of INDEL operation within InteractiveROSETTA using PDB 2AWJ. In the left window, the user selects the protein, anchor residues, a range of loop lengths, and the number of results desired. Additionally, the user can opt to retain the source sequence of loops used and/or enforce symmetry. Results are then listed in the table in the left window and can be viewed or saved before selecting one for further design

InteractiveROSETTA has previously been described in [23]. As a protocol within InteractiveROSETTA, INDEL may be invoked from the protocol menu on the left panel. From here the user selects all the parameters for the INDEL run, such as anchor residues and loop length. The resulting loops are then output in energy score order for the user to review. Each loop may be viewed before selecting one to design (Fig. 6).

INDEL can consistently find a loop with a low RMSD to the native loop when the loop length is constrained to the native, as long as the loop length is 12 or less (Fig. 2). For longer loops, the current database is not sufficiently complete to reliably return a low RMSD loop. Additionally, as the length of the loop inserted increases, so does the median RMSD of an inserted loop. The decreasing success rate with length is to be expected as the degrees of freedom of a loop increase with its sequence length. It is not likely that expanding the loop database by adding more known protein structures would help, since to improve the loop search the new proteins would have to contain new and different loop structures and novel loops appear increasingly rarely as the PDB expands. It might be possible to improve performance by allowing flexibility in the loop at the point of collision detection, but this would slow the response time.

In previously published experiments, Loophash [22], KIC [8], and Rosetta’s fragment-based loop builder [31] were used to insert a 12-residue loop in a 202-residue protein. Loophash takes 2 s, Rosetta 23 s, and KIC 260 s on average to perform these operations [22]. INDEL takes 10.6 s on average to insert a single loop. The slower constant-time search for INDEL versus Loophash is expected because INDEL searches the additional dimension of loop length.

## Conclusions

The new method provides fast/best solutions for loops of different lengths, and from there on an expert user makes the choice about which is the best loop and sequence to use. The user selection can then be refined with RosettaDesign or other tools (see Additional file 1: Figures S1–S9).

Our success in designing a fast lookup for variable length loops sets up the next challenge in variable-length protein design, that of energetic identification of the best loop and sequence. InteractiveRosetta already includes modules for protein design using fixed backbone (bbfix) and flexible backbone (KIC, backrub) approaches. As such, the approach to loop selection would be to apply a flexible backbone protein design script for each of the candidate loops, and select based on energy. The performance of energetic selection would be benchmarked using known engineered loops or natural indels of known structure.

In the T7 endoI linker-loop remodeling, expanding the search space to variable lengths was essential for success. The anchor residues of the loop corresponded to docked monomers on the phosphodiester backbone of PX DNA, instead of T7 endoI’s native substrate, the Holliday junction. Modeling experiments suggest that T7 endoI’s native-length linker peptide would be highly strained when T7 endoI is forced to bind PX DNA (unpublished). INDEL identified linker loops for T7 endoI that can better accommodate the PX DNA phosphodiester backbone conformation and potentially improve its specificity for PX DNA over Holliday junctions (Fig. 5).

This new tool enables the exploration of the space of insertions and deletions in the context of interactive protein design. The process could also be automated as a means to explore the ways a protein could evolve in length. To do this, we would need to establish a pipeline for energy minimization and protein design, but this is easily done in Rosetta (see Additional file 1). The resulting model could then cycle back through INDEL many times, producing an artificial evolutionary pathway.

## Availability and requirements

- **Project name:** InteractiveRosetta / INDEL
- **Project home page:** https://github.com/schenc3/InteractiveROSETTA/releases https://github.com/schenc3/InteractiveROSETTA/releases
- **Operating system(s):** Windows, macOS, Ubuntu Linux
- **Programming language:** Python/C++
- **Other requirements:** PyRosetta 3 (http://www.pyrosetta.org/dowhttp://www.pyrosetta.org/dow)
- **License:** GNU GPL v2.0
- **Any restrictions to use by non-academics:** PyRosetta license required for PyRosetta dependency

## Additional file

Additional file 1 Supplementary Figures for “Fast Design of Arbitrary Length Loops in Proteins Using InteractiveRosetta”. Storyboard walk-through of loop design using INDEL. (DOCX 3798 kb)

## Abbreviations

AT GFP: All-trans green fluorescent protein
HJ: Holliday junction
PX DNA: Paranemic crossover deoxyribonucleic acids
RMSD: Root mean squared deviation
T7 endoI: T7 endonuclease I
