# Supplementary material for: Fast design of arbitrary length loops in proteins using InteractiveRosetta
Source: BMC Bioinformatics. 2018 Sep 24;19:337. doi: 10.1186/s12859-018-2345-5 (PMC6154894; doi:10.1186/s12859-018-2345-5)
Supplement: Supplementary file 1 — Supplementary Figures for “Fast Design of Arbitrary Length Loops in Proteins Using InteractiveRosetta”. Storyboard walk-through of loop design using INDEL. (DOCX 3798 kb) [file 12859_2018_2345_MOESM1_ESM.docx]

**Supplementary Figures for “Fast Design of Arbitrary Length Loops in Proteins Using InteractiveRosetta” by Hooper et al.**

In the following series of figures, we walk you through the process of INDEL loop design within InteractiveRosetta, from loop search to side chain design to energy minimization.


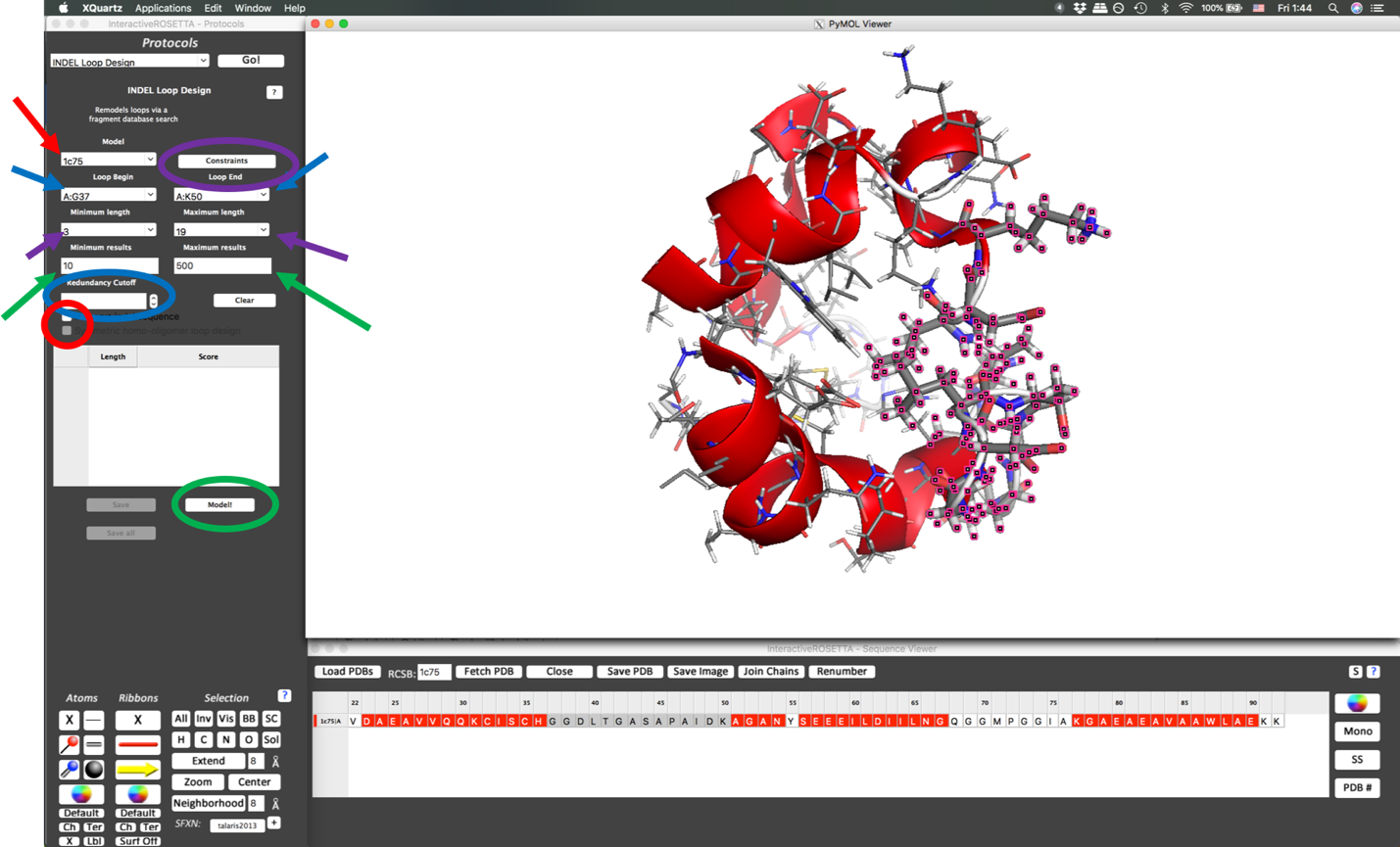


**Supplementary Figure 1.**

INDEL design begins in the INDEL Loop Design protocol. Once a protein is loaded for design, it is selected from the Model dropdown menu (red arrow). Loop anchor residues are then designated using the Loop Begin and Loop End menus (blue arrows); the desired loop will then be highlighted in the PyMOL and sequence viewers. The user then designates loop length (purple arrows), and desired amount of results (green arrows) via further dropdown menus. Additionally, a cutoff may be specified for redundant loops (blue circle), where smaller means loops can be more similar, or redundant. Constraints may be added to the loop design (purple circle), and the user may opt for symmetric homopolymer design or to maintain the sequence of the inserted loops (red circle). Design is initiated by pressing the **Model!** button (green circle).


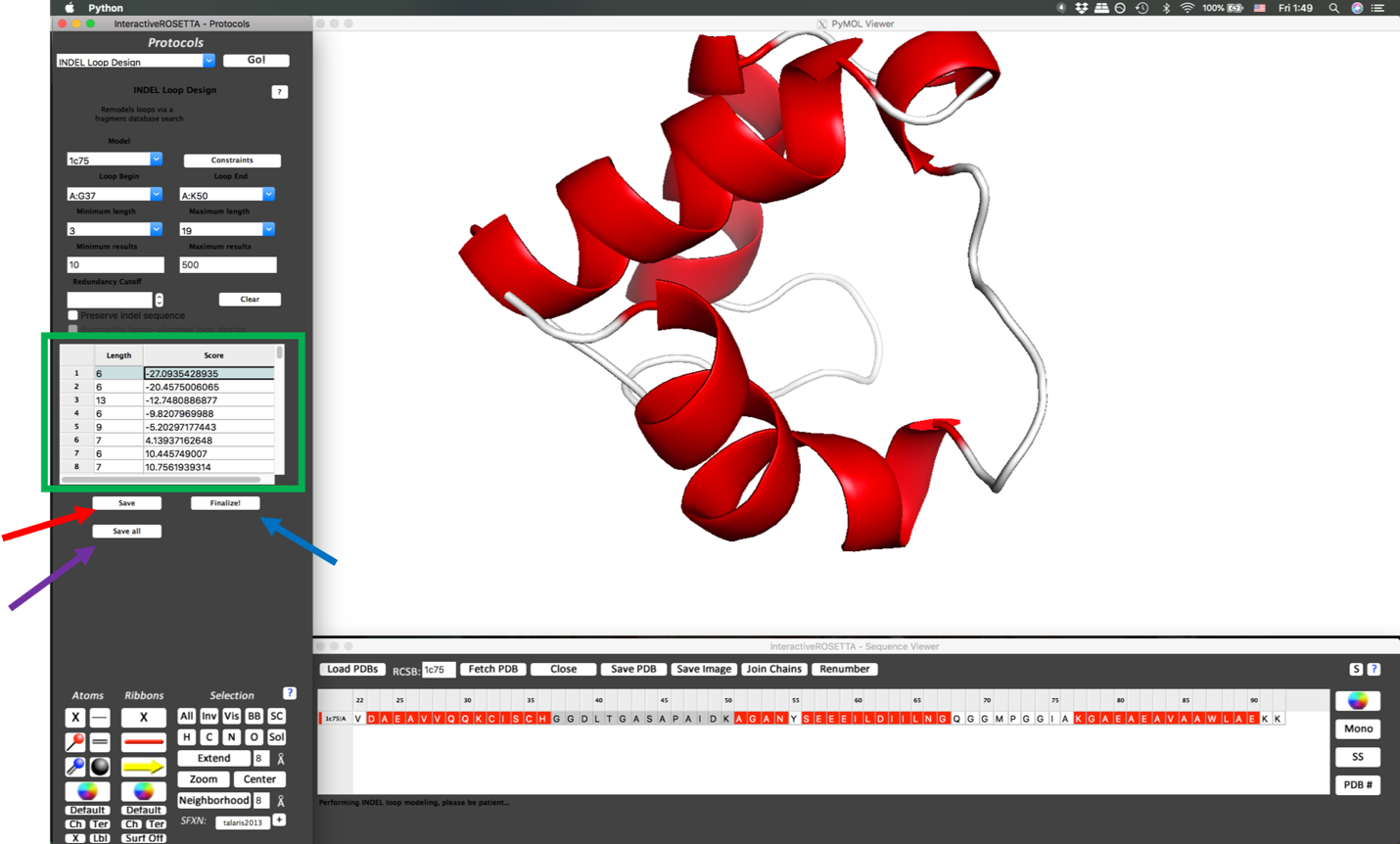


**Supplementary Figure 2.**

After completion of loop search and insertion, results are listed in the result table (green box). The highlighted entry is displayed in the PyMOL window. Each entry in the result table shows the length of the loop inserted as well as its total energy in REU (Rosetta energy units). The user may opt to save the highlighted entry (red arrow) or all results (purple arrow) before accepting one for further design via the **Finalize!** button (blue arrow).


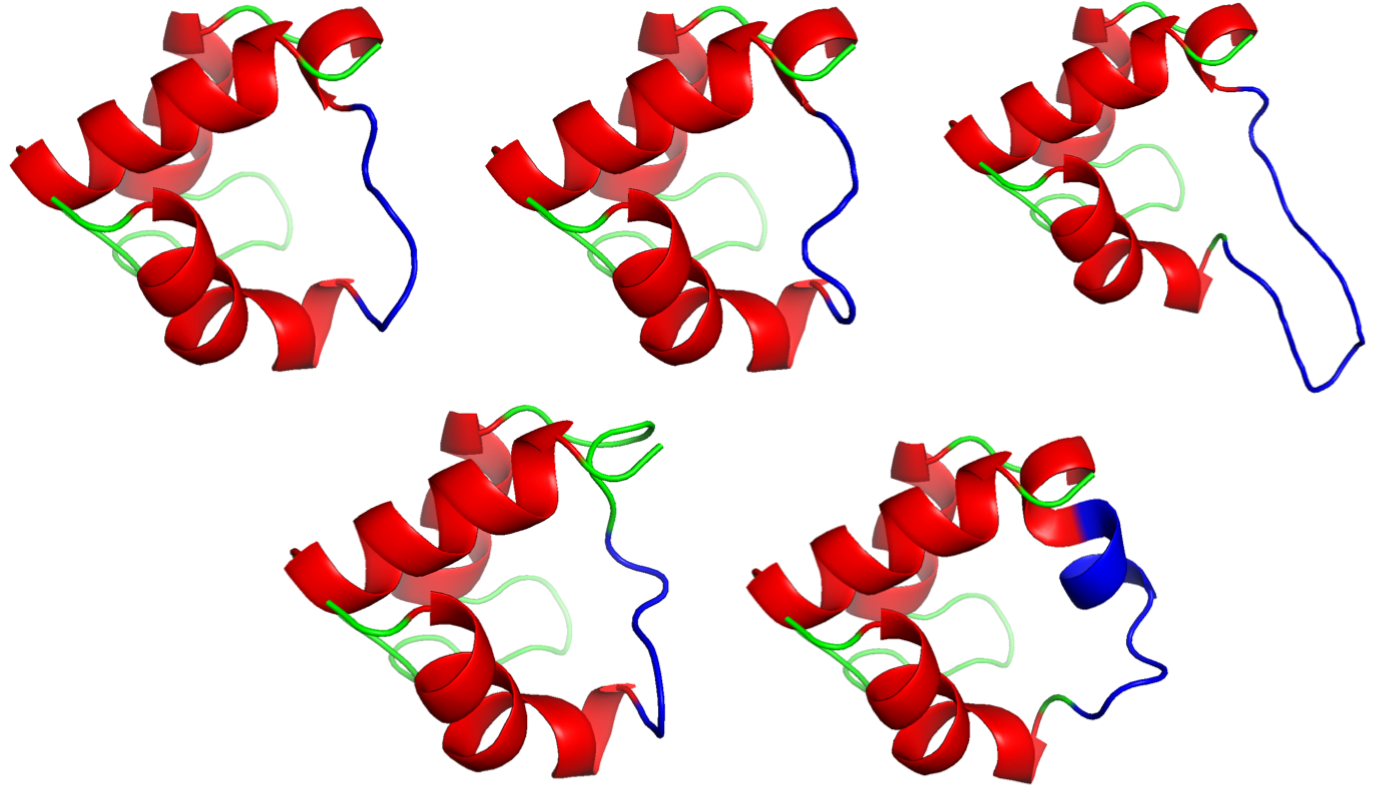


**Supplementary Figure 3.**

The top five lowest-scoring loops from INDEL are shown here with the inserted loops colored blue. In clockwise order from the top left, they are a 6 residue insertion with -27.09 REU, a 6 residue insertion with -20.46 REU, a 13 residue insertion with -12.75 REU, a 6 residue insertion with -9.82 REU, and a 9 residue insertion with -5.20 REU.


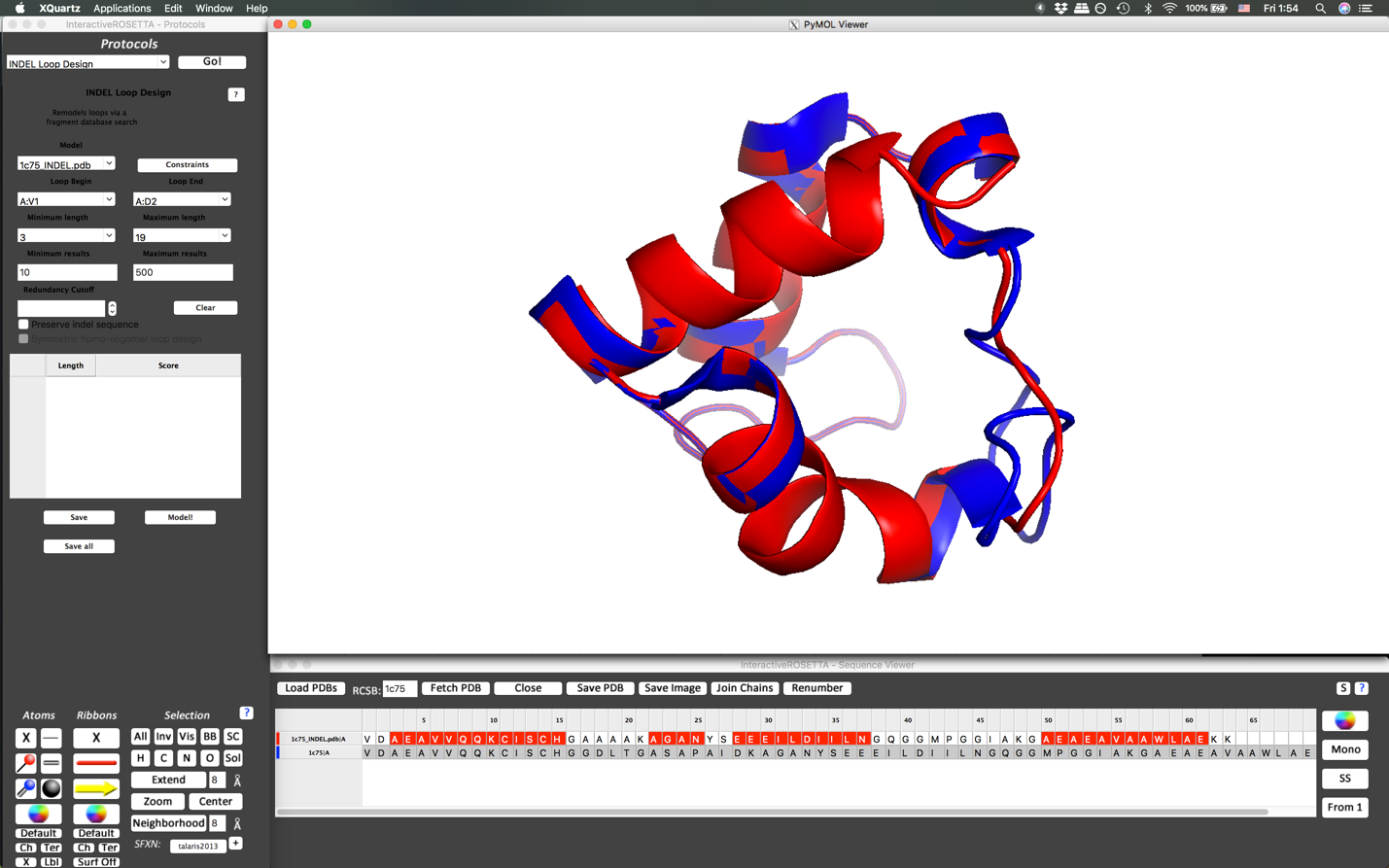


**Supplementary Figure 4.**

For comparison purposes, the original model structure may be reloaded (blue) and compared to the new structure (red).


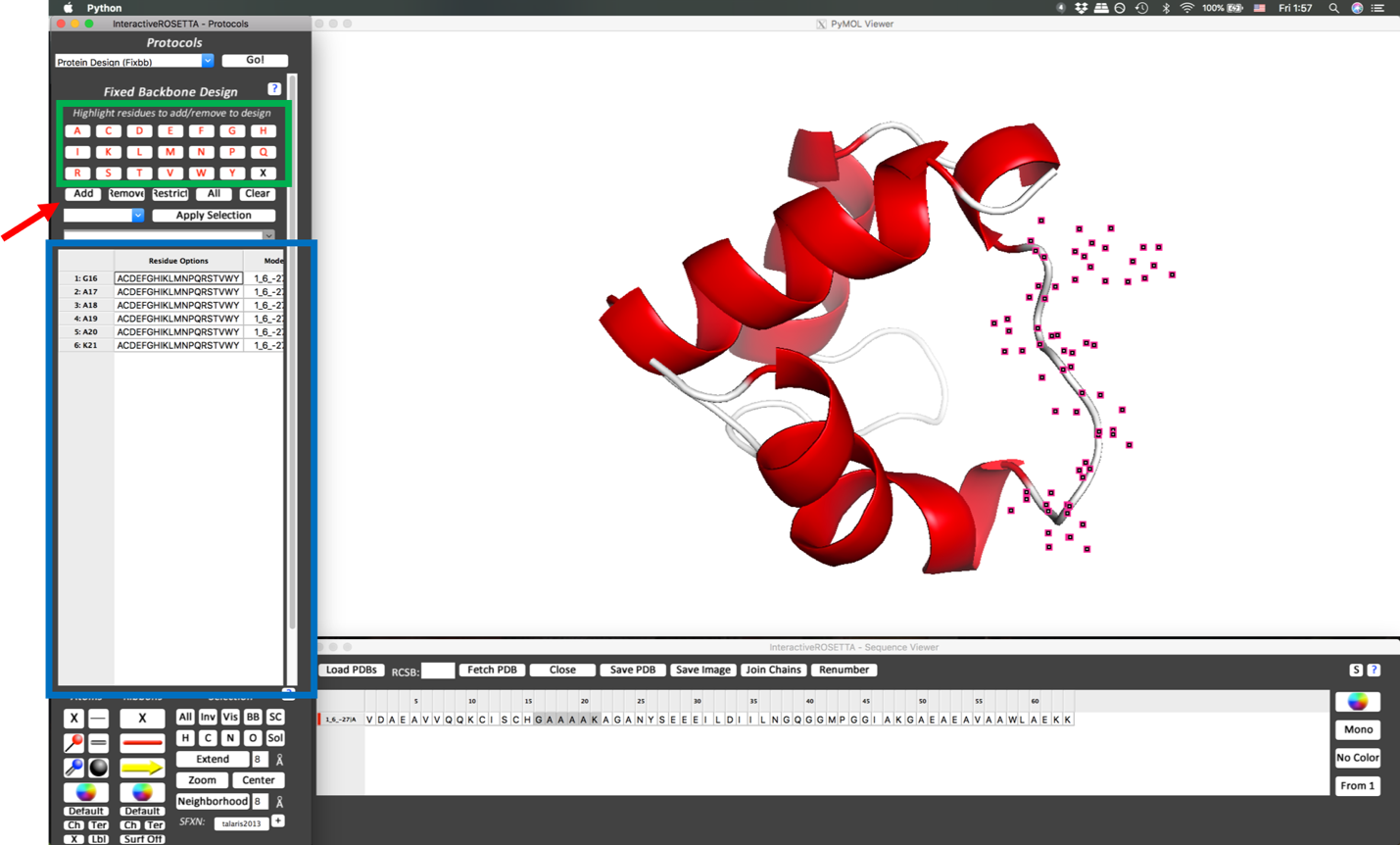


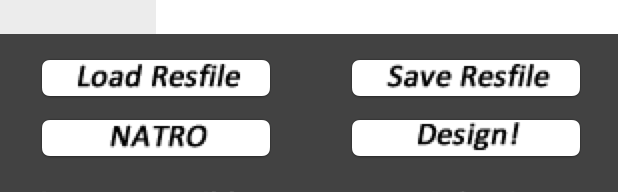


**Supplementary Figure 5.**

After INDEL, the loop of alanines inserted may be redesigned using the Protein Design (Fixbb) protocol. This protocol presents the user with a palette of residues (green box) where selected options are highlighted in red. The user then highlights the desired residues for redesign in the sequence viewer. By clicking the Add button (red arrow), the desired residues and design options are added and displayed in the table (blue box). These options may be saved to or loaded from a resfile via the appropriate buttons (accessible by scrolling down, shown in offset) before clicking the **Design!** button to initiate design. By default, all residues not selected will be kept as their native rotamers (NATRO). Toggling NATRO to NATAA searches all rotamers.


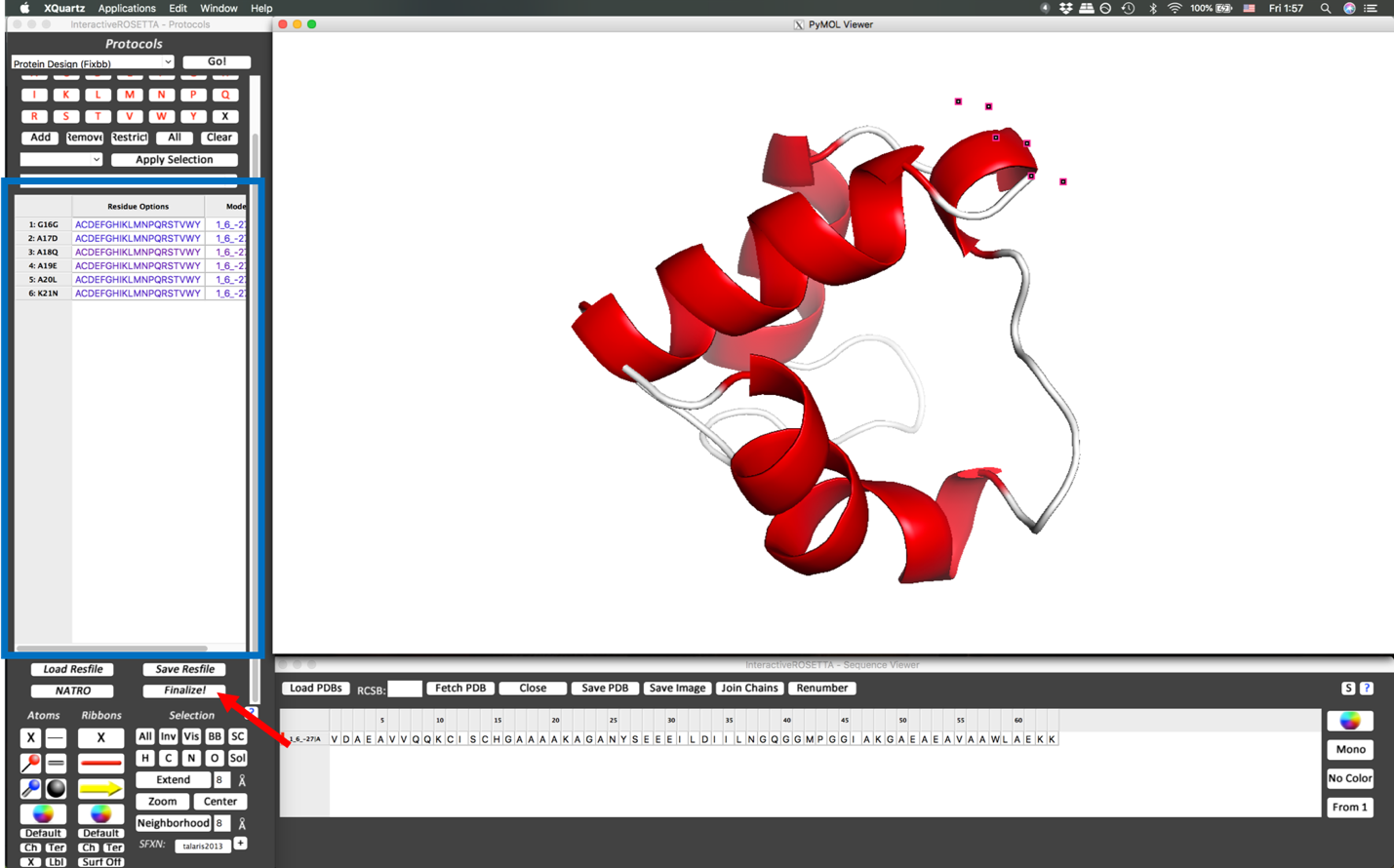


**Supplementary Figure 6.**

The results of design are output to the PyMOL viewer and the residue table where the entries are colored according to energy score (blue box, blue corresponds to low energy while red corresponds to high energy). The results can be accepted or rejected via the **Finalize!** button (red arrow).


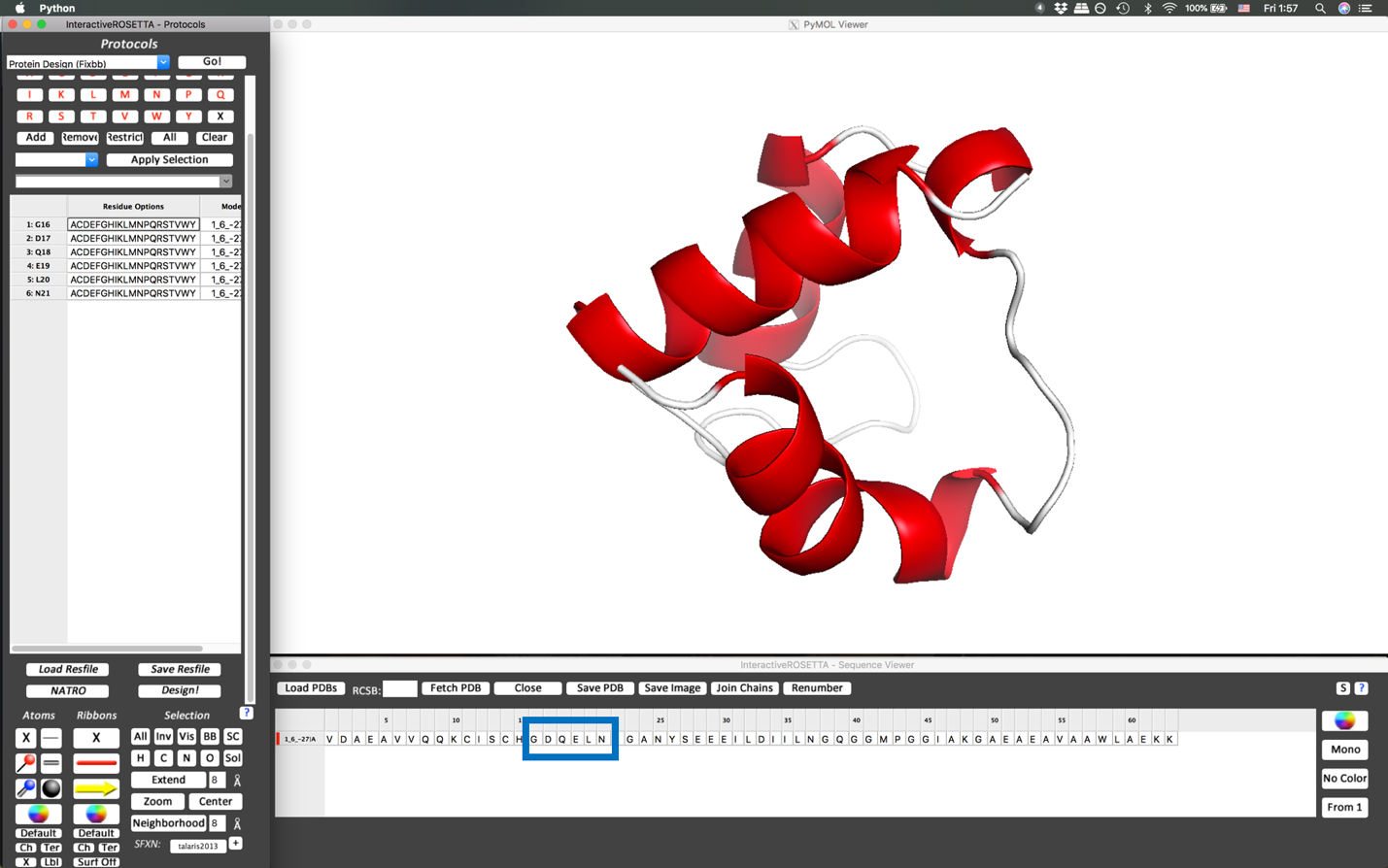


**Supplementary Figure 7.**

After finalization, the newly designed residues are displayed in the sequence viewer (blue box).


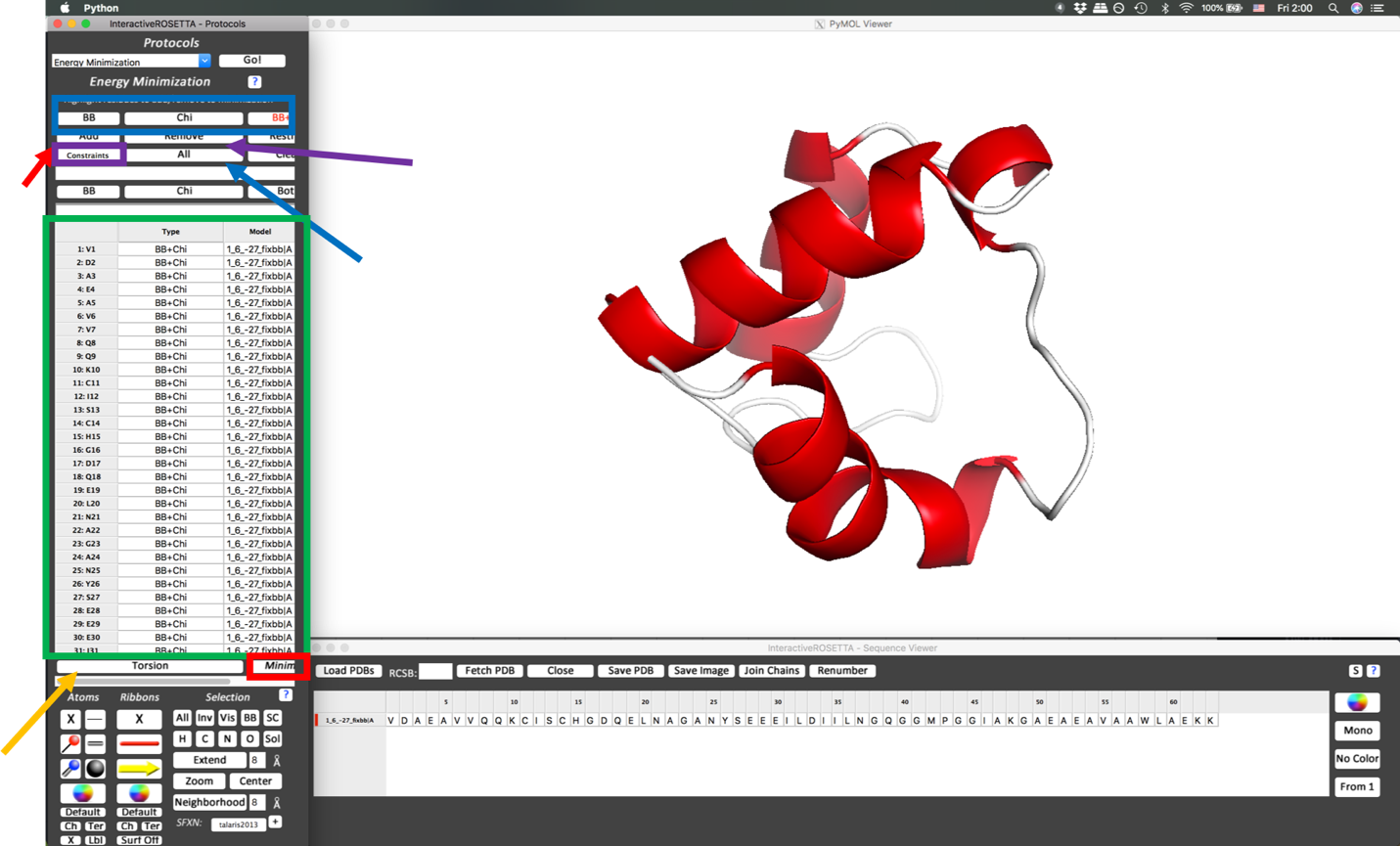


**Supplementary Figure 8.**

After design, the structure may be energy minimized using the Energy Minimization protocol. Here the user can elect to minimize backbone angles, chi angles, or both (blue box) with the selected option in red. Residues are added to the minimization by selection from the sequence viewer followed by clicking the Add button (red arrow). The All button may be used to add all residues (blue arrow). All residues added are shown in the residue table (green box). A residue may be highlighted and removed via the Remove button (purple arrow). Constraints may be added via the Constraints button (purple box). Torsion space or Cartesian space minimization may be toggled using the Torsion/Cartesian button (orange arrow). Minimization will begin once the Minimize button (red box) has been clicked.


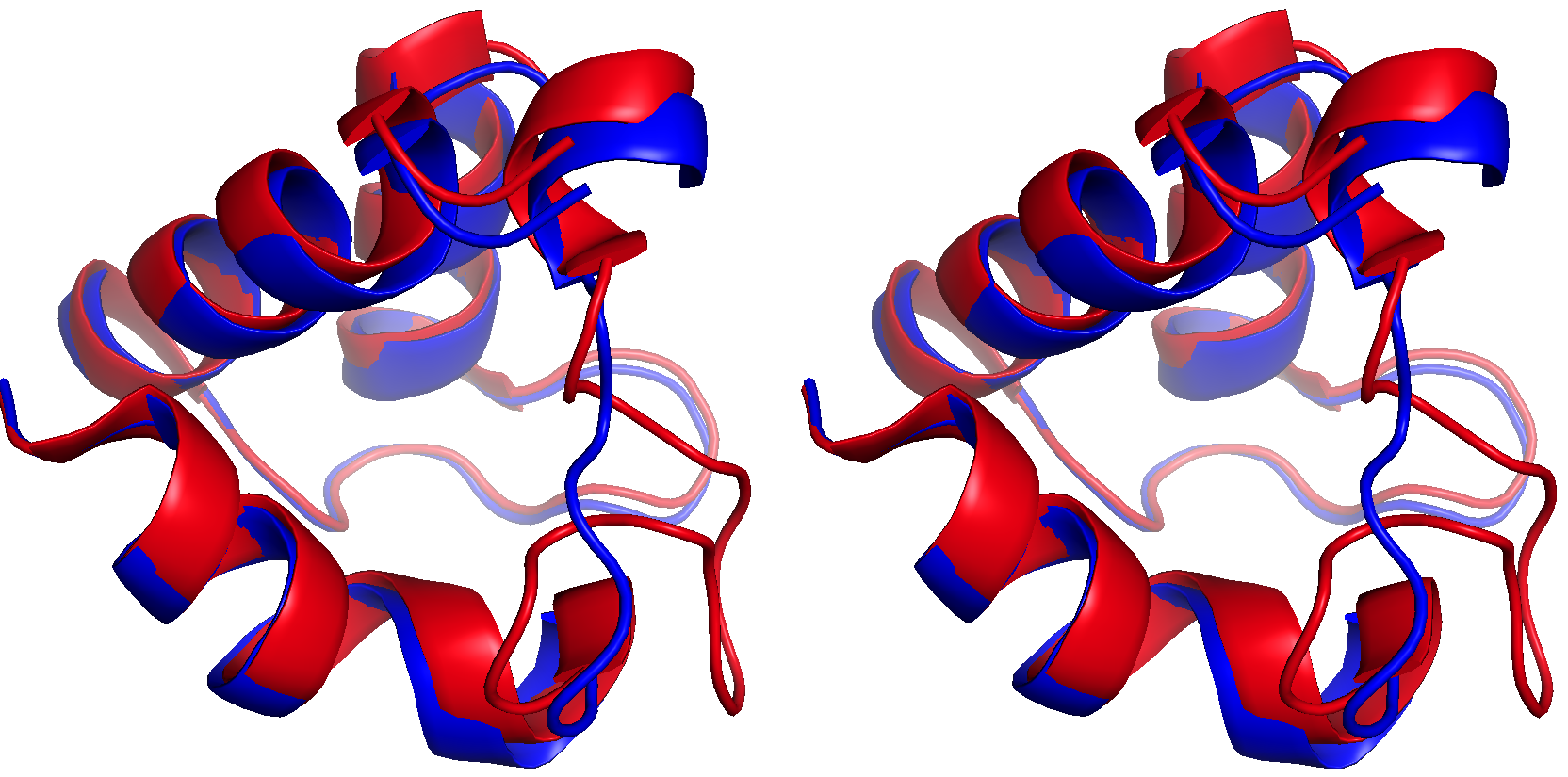


**Supplementary Figure 9.**

This walleye stereoimage shows both the original model (PDB 1C25, red) and the model after full INDEL redesign (blue).
